# Developing the engage for equity institutional multi-sector survey: Assessing academic institutional culture and climate for community-based participatory research (CBPR)

**DOI:** 10.1017/cts.2025.20

**Published:** 2025-02-05

**Authors:** Elizabeth Dickson, Alena Kuhlemeier, Prajakta Adsul, Shannon Sanchez-Youngman, Katie Myers, Tabia Henry Akintobi, Lisa G. Rosas, Jason A. Mendoza, John Oetzel, Paige Castro-Reyes, Christina Alaniz, Belkis Jacquez, Nina Wallerstein

**Affiliations:** 1 University of New Mexico Health Sciences Center, College of Population Health, Albuquerque, NM, USA; 2 University of New Mexico Health Sciences Center, Comprehensive Cancer Center, Albuquerque, NM, USA; 3 Full Circle Health Family Medicine Residency Program, Boise, ID, USA; 4 Morehouse School of Medicine, Prevention Research Center, Atlanta, GA, USA; 5 Stanford School of Medicine, Stanford, CA, USA; 6 University of Washington School of Medicine, Fred Hutchinson Cancer Center, Seattle, WA, USA; 7 The University of Waikato, New Zealand School of Management and Marketing, Hamilton, New Zealand; 8 Community-Campus Partnership for Health, Raleigh, NC, USA

**Keywords:** Patient- and community-engaged research, community-based participatory research, survey development, academic health center, CTSA

## Abstract

**Introduction:**

Community-engaged research/community-based participatory research/patient-engaged research (CEnR/CBPR/PEnR) are increasingly recognized as important approaches for addressing health equity. However, there is limited support for CEnR/CBPR/PEnR within Academic Health Centers (AHCs). It is important for AHCs to measure and monitor the context, process, and policies in support for CEnR/CBPR/PEnR. The *Engage for Equity (E2)* team developed the first Institutional Multi-Sector Survey (IMSS) instrument to assess and explore CEnR/CBPR/PEnR-related practices at three AHCs.

**Methods:**

Working with “champion teams” consisting of academic leaders, researchers, and patient/community partners at each AHC, we developed the IMSS to assess the following domains: institutional mission, vision, and values; CEnR/CBPR/PEnR policies/practices; community processes/structures; function of formal community advisory boards; climate/culture for CEnR/CBPR; perceptions of institutional leadership for CEnR/CBPR/PEnR. The survey was piloted to a convenience sample of CEnR/CBPR/PEnR participants at each AHC site.

**Results:**

A sample aggregated across all sites consisting of community (*n* = 49) and academic (*n* = 50) participants perceived high levels of advocacy for CEnR/CBPR/PEnR among their AHC research teams. Participants indicated that institutional leadership supported CEnR/CBPR/PEnR in principle, but resources to build CEnR/CBPR/PEnR capacity at their respective institutions were lacking. Differences in responses from community and academic partners are summarized.

**Conclusions:**

While limited by survey length and question adaptation, the findings contribute to identification of institutional barriers and facilitators to CEnR/CBPR/PEnR in AHCs. These findings are critically important to support and improve CEnR/CBPR/PEnR practice in academic institutions and to elevate community partner voices and needs for advancing community and patient partners’ research.

## Introduction

The growth of community-engaged research (CEnR) and community-based participatory research (CBPR) approaches to research in academic settings has exploded over several decades [1], with ongoing and significant impact from research centers supported by NIH-funded Clinical and Translational Science Awards [2]. CEnR/CBPR and even patient-engaged research (PEnR) have long since transitioned from an unexplored and often misunderstood orientation to research to an approach that funders have adopted, prioritized, and now mandate investigators and research institutions to employ with increasing expectations of engagement and health outcomes, as evidenced by the new National Academy of Medicine engagement model [3]. For investigators representing academic institutions who are already working with community or patient partners, these calls for action also demand increased authenticity within which partnering takes place including expanded accountability and transparency in all stages of the research process; amplification of power sharing, authority, resources, and decision-making; higher priority given to research concerns and needs of communities [4–7]. CBPR and CEnR have both been identified as research approaches to improve health equity [1,8,9], and expand the science of implementation [10–12]. However, within this context, many academic/community research partnerships operate within Academic Health Centers (AHC) where there is frequently a lack of organizational design and structure to adequately support CEnR, CBPR, and PEnR research partnerships [13,14]. The purpose of this paper is to introduce a tool for assessing the policies, practices, norms, and leadership that make up that institutional structure.

The *Engage for Equity (E2)* team from the University of New Mexico, Center for Participatory Research (UNM-CPR) along with numerous research partners [4], has received multiple stages of federal funding to develop and validate the CBPR conceptual model [15], identify CBPR promising practices [16], develop and psychometrically validate instruments measuring those promising practices [17], and test the *E2* intervention (which included a toolkit and measurement instruments) with over 200 funded research partnerships [4,18–21]. The *E2* intervention involves research partners (both academic and community) participating in multiple, facilitated workshops where they are introduced to the CBPR model, the partnership reflection tools, and exercises, which facilitate strategic planning and adoption of best partnering practices [21]. Through the course of several meetings, partners use survey data to identify their partnership’s unique strengths and challenges and, based on CBPR best practices, are able to strengthen their partnering practices. The *E2* intervention was originally developed for and tested at the *partnership level*, meaning groups consisting of investigators representing both academic institutions and community partners outside of academic institutions, working together on research projects within long-term or short-term partnerships [19]. As illustrated by the structural equation models by Oetzel and colleagues, the *E2* intervention had demonstrable impact on two pathways for how research partnerships meet their shared goals and outcomes: a formal structure pathway (formal agreements, community advisory boards, etc.) and a relationship structure pathway through partnership processes (trust building, conflict resolution, dialogue, leadership, etc.), both requiring a commitment to collective empowerment [17,22].

Participants in the *E2* intervention were able to identify specific contextual factors as structural barriers at their respective institutions in the form of policies, process, and reverse incentives preventing CEnR and CBPR [23]. It became clear that there was a need to 1) assess and identify what AHC academic and community partners needed to improve and strengthen their work together and 2) develop an intervention to strengthen institutional support for CEnR and CBPR approaches to research. In collaboration with members of the larger *E2* Think Tank (long-standing academic and community colleagues of the UNM Center for Participatory Research representing academic institutions and community organizations across the USA), the *E2* team proposed testing the feasibility of adapting the *E2* intervention, that successfully improved partnership-level outcomes, by scaling the intervention to the institutional AHC level and adding an additional element of *E2* coaching throughout the implementation process. This adaptation became known as *E2 Plus* and included five strategies: 1) conducting AHC institutional assessments; 2) identifying and creating institutional “champion teams” at each AHC site that included representation from both academic and community research partners; and 3) implementing the *E2* intervention (*E2* toolkit and workshops) with the institutional champion teams; 4) providing ongoing intervention guidance to each champion team, and 5) creating a multi-institution community of practice [24]. The champion teams at each institutional site consisted of 6–8 individuals, half of whom represented academic investigators and staff internal to the organization, half of whom represented community members and patient advocates external to the organization.

The *E2* Plus study aims were 1) assess institutional factors in 3 distinct AHCs to promote and sustain CBPR approaches; 2) implement the *E2* Plus intervention for champion teams at each AHC site and track team planning and institutional changes; 3) co-create community of practice for champion teams from each AHC site. The first study aim was to assess the institutional factors at each AHC site (e.g., capacity, structures, process, perceptions of commitment to CEnR). This involved the collection, analysis, and integration of both quantitative and qualitative data. The collection of quantitative data required the development of a unique survey tool with input and design guidance from all of the AHC champion teams. This study did not have hypothesis-driven research questions and as such, this paper will describe the development of the survey tool: the Institutional Multi-Sector Survey (IMSS) (please note that the tool was originally titled Institutional Multi-Stakeholder Survey, however the term stakeholder is no longer appropriate [25]). We will also summarize the data findings as part of the larger institutional assessment, present the aggregate findings of academic compared to community survey responses, describe how the findings were shared with the larger *E2* Community of Practice in testing of the IMSS tool, and discuss next steps. We conclude with learnings of the different views of academic and community partners who work within an AHC environment, yet how they both experience similar bureaucratic barriers to engagement, with similar limited knowledge of institutional policies in place to support CBPR/CEnR.

## Methods

The AHC sites recruited for this study were unique, geographically and demographically, with different patient and community populations served: 1) Morehouse School of Medicine’s Prevention Research Center, a private, Historically Black College/University located in Atlanta, Georgia; 2) Stanford University’s School of Medicine, Center for Clinical and Translational Research and Stanford Cancer Institute, a private, research-intensive (R1) institution in Palo Alto, California; and 3) the Fred Hutchinson Cancer Center’s Office of Community Outreach and Engagement, with University of Washington, a public R1 University, as well as two cancer care organizations, as a new cancer consortium in Seattle, Washington. Champion teams at each institutional site consisted of between 6 and 13 members representing both internal, academic spaces and external, community spaces.

### Survey tool development

Initially, we reviewed multiple bodies of literature that reviewed organization climate and collaborative governance processes that were supportive of CEnR and CBPR [14,26–29]. We adopted the validated measurement scales from the *E2* toolkit (Key Informant Survey [KIS] and Community Engagement Survey [CES]) [21] (https://engageforequity.org/tool_kit/surveys/) that assessed CEnR based on institutional structure, organizational culture and climate (i.e., norms, values, capacity for change), policies and practices (sharing financial practices, providing timely payments partners/subcontractors, tenure and promotion guidelines for CBPR), and partnerships processes (i.e., leadership, dialogue, conflict resolution, presence and role of community advisory boards). We asked for feedback on existing items and potentially missing items and constructs from project consultants, members of the *E2* Think Tank, and individuals recommended by the champion team leads. This feedback was integrated into the initial survey tool which was then reviewed with each of the AHC champion teams separately and then as a larger group to determine what additional institutional assessment constructs needed to be included in the survey of each AHC. Based on this feedback, additional sections were added to the survey instrument that assessed advocacy for patient/CEnR, perception of how top institutional leaders support CEnR/CBPR, and structural racism items (i.e., policies, practices, behaviors responsible for discrimination on race/ethnicity) adapted from a university-level climate survey tool from the University of Washington. The question items selected focused predominately on working conditions and practices internal to the AHC organization, as such the larger group of champion team members asked that the items on structural racism were only asked of those within the academic institution.

The champion team feedback also supported the decision to present survey questions for participants from two different institutional levels: 1) the larger, academic institutional level of the AHC, and 2) the level of community engagement centers *within* an AHC (CTSC, PRCs, offices of community engagement, etc.) representing the space where investigators work with community partners and have the most *relational* research interaction. While both investigators and institutional leaders can represent an academic institution to community, they also can become the *proxy* for an institution in their research partnerships with community partners. Collecting the data at both the institutional level and community engagement center level was key to identifying the perceived barriers and facilitators to CEnR/CBPR where individuals interacted as research partners. Survey questions were presented at the two different levels of the institutions: 1) specific to the characteristics of the institutional AHC level, and 2) specific to the community engagement center level with which community members were often more familiar.

A short set of demographic questions was included in the survey allowing participants to self-report age, gender, race/ethnicity, and if they were a community or academic partner. The final sections of the IMSS were:

1. Institutional mission, vision, and values statements (7 items)
2. Policies and practices to support equity-based CEnR/CBPR/PEnR (16 items)
3. Community processes and structures (6 items)
4. Formal community advisory boards (CAB) in the AHC (7 items)
5. Climate/culture for supporting equity-based CEnR/CBPR/PEnR (11 items)
6. Perceptions of top institutional leadership support of CEnR/CBPR/PEnR (4 items)
7. Structural racism (policies, practices, behaviors systematically discriminate based on race/ethnicity; item responses that were more inclusive of other forms of discrimination) (10 items)
8. Advocacy for CEnR/CBPR/PEnR (4 items)
9. Perceptions of CEnR/CBPR/PEnR (4 items)

The survey tool was finalized in English with members of the *E2* Think Tank reviewing the tool for content validity and to confirm that the survey items reflected data that could support CEnR/CBPR advocacy efforts with leadership in their respective organizations. The survey tool was uploaded to the UNM HSC REDCap [30] platform to support survey administration and ease of data management and protection and, after pilot testing, had an estimated completion time of 30 minutes. The final survey tool, recruitment protocol, and data collection and analysis protocols were approved by the UNM HSC Institutional Review Board (Protocol #21–320).

### Recruitment

Each champion team identified a convenience sample of institutional and community partners at their respective institutions with a recruitment goal of 50 participants at their respective AHC, including other researchers/investigators, staff, patients, community and patient advisory board members, institutional leaders and directors of finance, IRB, training, or research. Community members identified by champion team members were often connected to existing research projects. Recruitment emails were administered through REDCap with a unique survey link for each individual, and a $25 electronic gift card sent to each participant. A total of 3 reminders were sent to the email distribution list over 4 weeks.

### Analysis

Descriptive analyses of the IMSS sought to broadly assess the institutional context for CEnR/CBPR at the three AHCs. All data cleaning and analysis were performed in R v4.3.1 [31]. Based on experiences in delivering the intervention and associated a priori hypotheses regarding the potential differences between academic and community partners in terms of their perceptions of the AHC, all analyses were stratified by participants’ self-identified role (academic or community partner). Thus, we calculated frequencies of each Likert scale response and stratified all results by role. In the interest of transparency and in recognition of the important information conveyed by “Don’t know” responses, we have included these in our results. Significant differences between community and academic partners were tested in two ways. T-tests were conducted to determine significant differences between groups among Likert responses (e.g., “Strongly Disagree,” “Disagree,” “Neither Agree nor Disagree,” “Agree,” “Strongly Agree”), with “Don’t know” responses coded as missing. To assess significant differences in patterns of “Don’t know” responses compared to all other responses, we created new variables for each item that indicated whether a participant gave a substantive answer (coded as “0”) or responded “Don’t know” (coded as “1”). We then used Fisher’s exact test for categorical data and small sample sizes to assess significant differences between academic and community partners’ rates of “Don’t know” responses.

### Findings

From 150 invitations to participate in the survey, a total of 99 participants responded (66% response rate) to the IMSS across all 3 institutional sites (69.2% from University of Washington/Fred Hutchinson Cancer Center, 72.9% from Stanford School of Medicine, and 58% from Morehouse School of Medicine). The mean age of participants was 53 years, 61.6% identified as women, and 21.2% as men (17.2% did not provide their gender identification). Of the respondents that identified their race/ethnicity, participants identified as 2% American Indian/Alaska Native, 8.1% Asian, 29.3%, 27.3% Black/African American, 14.1% as Hispanic/Latino, 29.3% as White, and 4% as Multiple Races 4.0% (20% of participants did not provide their race/ethnicity). The representation of academic partners and community partners was almost evenly split, with 50.5% identifying as an academic partner and 49.5% as community partner (Table 1).

**Table 1.** Participant demographics

	%	N
Age	53.1	11.9
Sex/Gender identity		
Man	21.2%	21
Woman	61.6%	61
	17.2%	17
Race/Ethnicity (check all that apply)		
Asian	8.1%	8
Black/African American	27.3%	27
White	29.3%	29
Hispanic/Latino(a)	14.1%	14
American Indian/Alaska Native	2.0%	2
Multiple Races	4.0%	4
Missing	20.2%	20
Research partner		
Academic	50.5%	50
Community	49.5%	49

### Survey section results

The aggregated results across the 3 academic institutional sites from each of the 9 sections of the survey are summarized in Tables (2–7). Although the instrument collected data at the two institutional levels (institutional level and community engagement office level), analysis at those levels was not possible due to small sample sizes.

**Table 2.** Institutional policies and practices for communityengaged research (CEnR)

	Not at all		Slightly		Moderately		Often		All the time		Don’t know		*t*-test^a^ (p)	Fisher’s exact test^b^ (p)
Hiring and training patient and community partners as research coordinators/staff	12.9%	12	14.0%	13	9.7%	9	19.4%	18	10.8%	10	33.3%	31		
Academic	15.6%	7	24.4%	11	8.9%	4	15.6%	7	6.7%	3	28.9%	13	−2.36 (0.022)	(0.510)
Community	10.4%	5	4.2%	2	10.4%	5	22.9%	11	14.6%	7	37.5%	18		
Supporting involvement of patient and community partners in all stages of research (question to results)	5.3%	5	26.6%	25	10.6%	10	27.7%	26	11.7%	11	18.1%	17		
Academic	8.9%	4	40.0%	18	8.9%	4	17.8%	8	8.9%	4	15.6%	7	−3.25 (0.002)	(0.599)
Community	2.0%	1	14.3%	7	12.2%	6	36.7%	18	14.3%	7	20.4%	10		
Having policies that require partners to review grant applications for community benefit	19.1%	18	14.9%	14	6.4%	6	17.0%	16	9.6%	9	33.0%	31		
Academic	31.1%	14	24.4%	11	8.9%	4	4.4%	2	4.4%	2	26.7%	12	−4.94 (0.000)	(0.273)
Community	8.2%	4	6.1%	3	4.1%	2	28.6%	14	14.3%	7	38.8%	19		
Sharing information about financial practices with partners	19.4%	18	10.8%	10	11.8%	11	7.5%	7	7.5%	7	43.0%	40		
Academic	26.7%	12	13.3%	6	11.1%	5	2.2%	1	4.4%	2	42.2%	19	−2.60 (0.012)	(1.000)
Community	12.5%	6	8.3%	4	12.5%	6	12.5%	6	10.4%	5	43.8%	21		
Providing timely payments to community partners for participation in research	14.0%	13	9.7%	9	11.8%	11	20.4%	19	12.9%	12	31.2%	29		
Academic	22.7%	10	9.1%	4	18.2%	8	13.6%	6	9.1%	4	27.3%	12	−2.57 (0.013)	(0.505)
Community	6.1%	3	10.2%	5	6.1%	3	26.5%	13	16.3%	8	34.7%	17		
Providing timely payments to community subcontractors	11.7%	11	10.6%	10	12.8%	12	14.9%	14	11.7%	11	38.3%	36		
Academic	20.0%	9	15.6%	7	17.8%	8	13.3%	6	4.4%	2	28.9%	13	−3.56 (0.001)	(0.091)
Community	4.1%	2	6.1%	3	8.2%	4	16.3%	8	18.4%	9	46.9%	23		
Community engagement products in tenure and promotion guidelines for faculty	18.3%	17	9.7%	9	8.6%	8	12.9%	12	8.6%	8	41.9%	39		
Academic	26.7%	12	17.8%	8	11.1%	5	6.7%	3	4.4%	2	33.3%	15	−3.30 (0.002)	(0.141)
Community	10.4%	5	2.1%	1	6.3%	3	18.8%	9	12.5%	6	50.0%	24		
IRB policies and practices that support CEnR	10.8%	10	17.2%	16	8.6%	8	11.8%	11	21.5%	20	30.1%	28		
Academic	17.8%	8	24.4%	11	15.6%	7	8.9%	4	8.9%	4	24.4%	11	−4.26 (0.000)	(0.268)
Community	4.2%	2	10.4%	5	2.1%	1	14.6%	7	33.3%	16	35.4%	17		
Minimizing barriers to participation for partners in research	13.0%	12	20.7%	19	17.4%	16	13.0%	12	14.1%	13	21.7%	20		
Academic	13.3%	6	31.1%	14	24.4%	11	4.4%	2	6.7%	3	20.0%	9	−2.82 (0.006)	(0.802)
Community	12.8%	6	10.6%	5	10.6%	5	21.3%	10	21.3%	10	23.4%	11		
Having written standards that provide expectations around CEnR	20.4%	19	8.6%	8	9.7%	9	15.1%	14	14.0%	13	32.3%	30		
Academic	31.1%	14	13.3%	6	11.1%	5	6.7%	3	6.7%	3	31.1%	14	−3.99 (0.000)	(0.828)
Community	10.4%	5	4.2%	2	8.3%	4	22.9%	11	20.8%	10	33.3%	16		
Evaluating if staff/faculty meet expectations for CEnR	25.3%	23	11.0%	10	4.4%	4	14.3%	13	6.6%	6	38.5%	35		
Academic	38.6%	17	20.5%	9	0.0%	0	11.4%	5	2.3%	1	27.3%	12	−3.56 (0.001)	(0.051)
Community	12.8%	6	2.1%	1	8.5%	4	17.0%	8	10.6%	5	48.9%	23		
Offering education for partners on research processes	11.8%	11	18.3%	17	12.9%	12	19.4%	18	18.3%	17	19.4%	18		
Academic	11.1%	5	31.1%	14	15.6%	7	15.6%	7	6.7%	3	20.0%	9	−3.02 (0.003)	(1.000)
Community	12.5%	6	6.3%	3	10.4%	5	22.9%	11	29.2%	14	18.8%	9		
Having standards for oral and written information that meets cultural, linguistic, and literacy needs of community	8.7%	8	16.3%	15	15.2%	14	19.6%	18	13.0%	12	27.2%	25		
Academic	11.1%	5	26.7%	12	17.8%	8	15.6%	7	8.9%	4	20.0%	9	−2.53 (0.014)	(0.162)
Community	6.4%	3	6.4%	3	12.8%	6	23.4%	11	17.0%	8	34.0%	16		
Identifying and adapting to changes within communities	9.8%	9	13.0%	12	17.4%	16	25.0%	23	14.1%	13	20.7%	19		
Academic	13.6%	6	22.7%	10	22.7%	10	13.6%	6	6.8%	3	20.5%	9	−3.80 (0.000)	(1.000)
Community	6.3%	3	4.2%	2	12.5%	6	35.4%	17	20.8%	10	20.8%	10		
Having community input in strategic planning at leadership level	16.1%	15	16.1%	15	12.9%	12	14.0%	13	12.9%	12	28.0%	26		
Academic	24.4%	11	24.4%	11	20.0%	9	4.4%	2	4.4%	2	22.2%	10	−4.38 (0.000)	(0.257)
Community	8.3%	4	8.3%	4	6.3%	3	22.9%	11	20.8%	10	33.3%	16		
Having funding strategies to mobilize community partners to research health inequities	10.9%	10	17.4%	16	19.6%	18	15.2%	14	9.8%	9	27.2%	25		
Academic	15.9%	7	29.5%	13	18.2%	8	9.1%	4	4.5%	2	22.7%	10	−3.55 (0.000)	(0.482)
Community	6.3%	3	6.3%	3	20.8%	10	20.8%	10	14.6%	7	31.3%	15		

^a^t-tests assessed differences between community and academic partners on all scale levels, excluding “don’t know” responses.^b^Fisher’s exact tests assessed differences between community and academic partners on “don’t know” response patterns (don’t know vs. any other response).

**Table 3.** Community processes and structures

	Not at all		To a small extent		To a moderate extent		To a great extent		To a complete extent		Don’t know		*t*-test^a^ (p)	Fisher’s exact test^b^ (p)
To what extent are community members engaged in strategic planning at the top institutional level	17.4%	16	19.6%	18	5.4%	5	7.6%	7	6.5%	6	43.5%	40		
Academic	24.4%	11	22.2%	10	4.4%	2	8.9%	4	4.4%	2	35.6%	16	−1.37 (0.177)	(0.147)
Community	10.6%	5	17.0%	8	6.4%	3	6.4%	3	8.5%	4	51.1%	24		
To what extent are patients engaged in strategic planning at the top institutional level	22.8%	21	18.5%	17	3.3%	3	3.3%	3	5.4%	5	46.7%	43		
Academic	31.1%	14	22.2%	10	4.4%	2	2.2%	1	4.4%	2	35.6%	16	−1.25 (0.220)	(0.040)
Community	14.9%	7	14.9%	7	2.1%	1	4.3%	2	6.4%	3	57.4%	27		
	Strongly disagree		Somewhat disagree		Neither agree nor disagree		Somewhat agree		Strongly agree		Don’t know			
Your institution is recognized for its reputation in community and patient-engaged research	15.4%	14	17.6%	16	7.7%	7	19.8%	18	34.1%	31	5.5%	5		
Academic	20.5%	9	25.0%	11	11.4%	5	18.2%	8	22.7%	10	2.3%	1	−2.78 (0.007)	(0.362)
Community	10.6%	5	10.6%	5	4.3%	2	21.3%	10	44.7%	21	8.5%	4		
External stakeholders who engage with leadership at your institution represent interests of community residents	5.6%	5	15.6%	14	7.8%	7	26.7%	24	23.3%	21	21.1%	19		
Academic	6.8%	3	25.0%	11	11.4%	5	27.3%	12	11.4%	5	18.2%	8	−3.18 (0.002)	(0.608)
Community	4.3%	2	6.5%	3	4.3%	2	26.1%	12	34.8%	16	23.9%	11		
Your institution is recognized for its reputation in equity-based research	13.2%	12	18.7%	17	9.9%	9	18.7%	17	29.7%	27	9.9%	9		
Academic	18.2%	8	29.5%	13	13.4%	6	11.4%	5	25.0%	11	2.3%	1	−2.75 (0.007)	(0.031)
Community	8.5%	4	8.5%	4	6.4%	3	25.5%	12	34.0%	16	17.0%	8		
Community-engaged research included in criteria on which faculty at your institution are evaluated/promoted	18.0%	16	12.4%	11	7.9%	7	6.7%	6	13.5%	12	41.6%	37		
Academic	27.9%	12	23.3%	10	14.0%	6	9.3%	4	7.0%	3	18.6%	8	−2.81 (0.009)	(0.000)
Community	8.7%	4	2.2%	1	2.2%	1	4.3%	2	19.6%	9	63.0%	29		

^a^t-tests assessed differences between community and academic partners on all scale levels, excluding “don’t know” responses. ^b^Fisher’s exact tests assessed differences between community and academic partners on “don’t know” response patterns (don’t know vs. any other response).

**Table 4.** Formal Community Advisory Boards (CABs)

CAB engages in the following practices:	Not at all		To a small extent		To a moderate extent		To a great extent		To a complete extent		Don’t know		*t*-test^a^ (p)	Fisher’s exact test^b^ (p)
Identifies research needs and priorities	2.2%	1	13.3%	6	20.0%	9	31.1%	14	15.6%	7	17.8%	8		
Academic	0.0%	0	16.7%	4	25.0%	6	16.7%	4	16.7%	4	25.0%	6	−0.526 (0.602)	(0.252)
Community	4.8%	1	9.5%	2	14.3%	3	47.6%	10	14.3%	3	9.5%	2		
Consults on cultural issues	2.3%	1	6.8%	3	11.4%	5	47.7%	21	18.2%	8	13.6%	6		
Academic	4.3%	1	8.7%	2	13.0%	3	43.5%	10	13.0%	3	17.4%	4	−1.39 (0.174)	(0.666)
Community	0.0%	0	4.8%	1	9.5%	2	52.4%	10	23.8%	5	9.5%	2		
Strengthens collaborations between academic and community/patient partners	2.3%	1	4.5%	2	18.2%	8	47.7%	21	20.5%	9	6.8%	3		
Academic	4.3%	1	4.3%	1	21.7%	5	43.5%	10	13.0%	3	13.0%	3	−1.41 (0.167)	(0.234)
Community	0.0%	0	4.8%	1	14.3%	3	52.4%	11	28.6%	6	0.0%	0		
Provides ideas/assistance for research subjects/participant recruitment	2.3%	1	4.5%	2	22.7%	10	40.9%	18	15.9%	7	13.6%	6		
Academic	4.2%	1	8.3%	2	20.8%	5	33.3%	8	12.5%	3	20.8%	5	−1.43 (0.163)	(0.198)
Community	0.0%	0	0.0%	0	25.0%	5	50.0%	10	20.0%	4	5.0%	1		
Develops plans for using findings to benefit the community	7.1%	3	9.5%	4	11.9%	5	38.1%	16	19.1%	8	14.3%	6		
Academic	8.7%	2	13.0%	3	13.0%	3	30.4%	7	8.7%	2	26.1%	6	−1.82 (0.078)	(0.024)
Community	5.3%	1	5.3%	1	10.5%	2	47.4%	9	31.6%	6	0.0%	0		
Assists with sustainability planning	6.8%	3	11.4%	5	18.2%	8	29.5%	13	9.1%	4	25.0%	11		
Academic	4.2%	1	16.7%	4	25.0%	6	8.3%	2	8.3%	2	37.5%	9	−1.39 (0.174)	(0.044)
Community	10.0%	2	5.0%	1	10.0%	2	55.0%	11	10.0%	2	10.0%	2		
Assists with strategic planning	7.0%	3	9.3%	4	14.0%	6	39.5%	17	16.3%	7	14.0%	6		
Academic	8.7%	2	0.0%	0	26.1%	6	26.1%	6	21.7%	5	17.4%	4	0.34 (0.737)	(0.669)
Community	5.0%	1	20.0%	4	0.0%	0	55.0%	11	10.0%	2	10.0%	2		

^a^t-tests assessed differences between community and academic partners on all scale levels, excluding “don’t know” responses.^b^Fisher’s exact tests assessed differences between community and academic partners on “don’t know” response patterns (don’t know vs. any other response).

**Table 5.** Climate and culture for supporting equity-based community-engaged research (CEnR)

	Strongly disagree		Somewhat disagree		Neither agree nor disagree		Somewhat agree		Strongly agree		Don’t know		*t*-test^a^ (p)	Fisher’s exact test^b^ (p)
Researchers/staff are willing to change how research is conducted in response to patient/community feedback.	1.6%	1	7.9%	5	4.8%	3	27.0%	17	50.8%	32	7.9%	5		
Academic	0	0	6.5%	2	6.5%	2	32.3%	10	48.4%	15	6.5%	2	0.25 (0.800)	(1.000)
Community	3.1%	1	9.4%	3	3.1%	1	21.9%	7	53.1%	17	9.4%	3		
Researchers/staff regularly evaluate how to improve work	1.6%	1	4.8%	3	6.3%	4	33.3%	21	30.2%	19	23.8%	15		
Academic	0.0%	0	1.6%	1	12.9%	4	45.2%	14	25.8%	8	12.9%	4	−0.39 (0.696)	(0.075)
Community	3.1%	1	6.3%	2	0.0%	0	21.9%	7	34.4%	11	34.4%	11		
Staff frustration is common	11.1%	7	9.5%	6	11.1%	7	14.3%	9	14.3%	9	39.7%	25		
Academic	12.9%	4	9.7%	3	12.9%	4	16.1%	5	16.1%	5	32.3%	10	0.03 (0.977)	0.86 (0.306)
Community	9.4%	3	9.4%	3	9.4%	3	12.5%	4	12.5%	4	46.9%	15		
Researchers/staff regularly reflect on working towards equity	4.8%	3	8.1%	5	8.1%	5	33.9%	21	32.3%	20	12.9%	8		
Academic	6.5%	2	6.5%	2	9.7%	3	45.2%	14	29.0%	9	3.2%	1	−0.41 (0.682)	(0.053)
Community	3.2%	1	9.7%	3	6.5%	2	22.6%	7	35.5%	11	22.6%	7		
Office/Center of Engagement mobilizes community partnerships to address social, environmental, and economic conditions influencing health outcomes.	1.6%	1	6.3%	4	3.2%	2	30.2%	19	54.0%	34	4.8%	3		
Academic	0.0%	0	9.7%	3	3.2%	1	35.5%	11	45.2%	14	6.5%	2	−0.85 (0.398)	(0.613)
Community	3.1%	1	3.1%	1	3.1%	1	25.0%	8	62.5%	20	3.1%	1		
Office/Center of Engagement works with community partners to support programs/interventions to address health inequities.	3.2%	2	3.2%	2	6.5%	4	30.6%	19	53.2%	33	3.2%	2		
Academic	3.3%	1	3.3%	1	3.3%	1	40.0%	12	16.7%	14	3.3%	1	−0.31 (0.758)	(1.000)
Community	3.1%	1	3.1%	1	9.4%	3	21.9%	7	59.4%	19	3.1%	1		
Office/Center of Engagement advocates for policies addressing health inequities.	6.5%	4	3.2%	2	14.5%	9	25.8%	16	43.5%	27	6.5%	4		
Academic	10.0%	3	6.7%	2	16.7%	5	26.7%	8	33.3%	10	6.7%	2	−2.02 (0.049)	(1.000)
Community	3.1%	1	0.0%	0	12.5%	4	25.0%	8	53.1%	17	6.3%	2		
Office/Center of Engagement contributes to new insights, evidence base, and innovative solutions to address health inequities.	1.6%	1	1.6%	1	6.3%	4	31.3%	20	56.3%	36	3.1%	2		
Academic	0.0%	0	3.2%	1	0.0%	0	41.9%	13	54.8%	17	0.0%	0	0.46 (0.647)	(0.493)
Community	3.0%	1	0.0%	0	12.1%	4	21.2%	7	57.6%	19	6.1%	2		
Office/Center of Engagement has the budget/financial resources to support community/patient-engaged research	17.2%	11	10.9%	7	7.8%	5	21.9%	14	14.1%	9	28.1%	18		
Academic	22.6%	7	16.1%	5	9.7%	3	25.8%	8	6.5%	2	19.4%	6	−1.73 (0.091)	(0.169)
Community	12.1%	4	6.1%	2	6.1%	2	18.2%	6	21.2%	7	36.4%	12		
Office/Center of Engagement has training resources to support community/patient-engaged research	9.5%	6	19.0%	12	3.2%	2	23.8%	15	23.8%	15	20.6%	13		
Academic	6.7%	2	36.7%	11	3.3%	1	30.0%	9	10.0%	3	13.3%	4	−2.21 (0.032)	(0.221)
Community	12.1%	4	3.0%	1	3.0%	1	18.2%	6	36.4%	12	27.3%	9		
Office/Center of Engagement has the necessary staffing resources to support community/patient-engaged research	18.8%	12	18.8%	12	7.8%	5	18.8%	12	14.1%	9	21.9%	14		
Academic	22.6%	7	32.3%	10	9.7%	3	16.1%	5	3.2%	1	16.1%	5	−2.81 (0.007)	(0.369)
Community	15.2%	5	6.1%	2	6.1%	2	21.2%	7	24.2%	8	27.3%	9		

^a^t-tests assessed differences between community and academic partners on all scale levels, excluding “don’t know” responses.^b^Fisher’s exact tests assessed differences between community and academic partners on “don’t know” response patterns (don’t know vs. any other response).

**Table 6.** Perceptions of leadership engagement in supporting community-engaged research (CEnR)

	Strongly disagree		Somewhat disagree		Neither agree nor disagree		Somewhat agree		Strongly agree		Don’t know		*t*-test^a^ (p)	Fisher’s exact test^b^ (p)
Ensures researchers and staff have resources necessary to conduct CEnR	9.1%	8	21.6%	19	5.7%	5	18.2%	16	15.9%	14	29.5%	26		
Academic	16.3%	7	32.6%	14	7.0%	3	18.6%	8	11.6%	5	14.0%	6	−3.04 (0.003)	(0.002)
Community	2.2%	1	11.1%	5	4.4%	2	17.8%	8	20.0%	9	44.4%	20		
Strongly supports training and development of community-engaged scholars	6.9%	6	19.5%	17	12.6%	11	16.1%	14	24.1%	21	20.7%	18		
Academic	9.3%	4	34.9%	15	11.6%	5	16.3%	7	16.3%	7	11.6%	5	−3.19 (0.002)	(0.062)
Community	4.5%	2	4.5%	2	13.6%	6	15.9%	7	31.8%	14	29.5%	13		
Supports researchers and staff to learn from community partners	6.9%	6	13.8%	12	10.3%	9	26.4%	23	18.4%	16	24.1%	21		
Academic	9.5%	4	23.8%	10	16.7%	7	23.8%	10	11.9%	5	14.3%	6	−3.05 (0.003)	(0.047)
Community	4.4%	2	4.4%	2	4.4%	2	28.9%	13	24.4%	11	33.3%	15		
Encourages researchers to engage in health equity research	3.4%	3	5.7%	5	12.5%	11	25.0%	22	34.1%	30	19.3%	17		
Academic	4.7%	2	9.3%	4	14.0%	6	34.9%	15	32.6%	14	4.7%	2	−1.31 (0.195)	(0.001)
Community	2.2%	1	2.2%	1	11.1%	5	15.6%	7	35.6%	16	33.3%	15		

at-tests assessed differences between community and academic partners on all scale levels, excluding “don’t know” responses.^b^Fisher’s exact tests assessed differences between community and academic partners on “don’t know” response patterns (don’t know vs. any other response).

**Table 7.** Advocacy for and perceptions of community-engaged research (CEnR) or patient-engaged research

	Strongly disagree		Somewhat disagree		Neither agree nor disagree		Somewhat agree		Strongly agree		Don’t know		*t*-test^a^ (p)	Fisher’s exact test^b^ (p)
	%	N	%	N	%	N	%	N	%	N	%	N		
I am, or researchers I work with, are able to reflect and identify systems of power that influence treatment of community members and patients	3.5%	3	7.1%	6	14.1%	12	34.1%	29	32.9%	28	8.2%	7		
Academic	2.4%	1	9.8%	4	14.6%	6	43.9%	18	24.4%	10	4.9%	2	−0.94 (0.351)	(0.435)
Community	4.5%	2	4.5%	2	13.6%	6	25.0%	11	40.9%	18	11.4%	5		
I am, or researchers I work with, are able to negotiate for equitable services and research on behalf of community members/patients	5.8%	5	11.6%	10	16.3%	14	27.9%	24	24.4%	21	14.0%	12		
Academic	7.1%	3	14.2%	6	16.7%	7	38.1%	16	14.3%	6	9.5%	4	−1.46 (0.150)	(0.353)
Community	4.5%	2	9.1%	4	15.9%	7	18.2%	8	34.1%	15	18.2%	8		
I, or the researchers I work with, evaluate the effectiveness of efforts to address racial equity issues that impact community members and patients.	4.7%	4	5.9%	5	14.1%	12	38.8%	33	28.2%	24	8.2%	7		
Academic	4.9%	2	7.3%	3	14.6%	6	51.2%	21	19.5%	8	2.4%	1	−1.01 (0.314)	(0.111)
Community	4.5%	2	4.5%	2	13.6%	6	27.3%	12	36.6%	16	13.6%	6		
I am, or the researchers I work with, are comfortable developing an action plan to confront barriers to health equity that impacts community members and patients.	1.2%	1	4.7%	4	15.1%	13	32.6%	28	38.4%	33	8.1%	7		
Academic	0.0%	0	7.1%	3	16.7%	7	38.1%	16	35.7%	15	2.4%	1	−0.63 (0.530)	(0.110)
Community	2.3%	1	2.3%	1	13.6%	6	27.3%	12	40.9%	18	13.6%	6		
My research, or the research I work with is not relevant to community stakeholders	71.2%	62	14.9%	13	2.3%	2	3.4%	3	2.3%	2	5.7%	5		
Academic	71.4%	30	26.2%	11	2.4%	2	0.0%	0	0.0%	0	0.0%	0	−1.06 (0.292)	(0.056)
Community	71.1%	32	4.4%	2	2.2%	1	6.7%	3	4.4%	2	11.1%	5		
I, or the researchers I work with, have limited access to/knowledge of community stakeholders	46.5%	41	17.4%	15	9.3%	8	15.1%	13	4.7%	4	7.0%	6		
Academic	46.3%	19	22.0%	9	12.2%	5	17.1%	7	2.4%	1	0.0%	0	−0.01 (0.990)	(0.027)
Community	46.7%	21	13.3%	6	6.7%	3	13.3%	6	6.7%	3	13.3%	6		
I, or the researchers I work with, have limited knowledge of or experience engaging community stakeholders	44.8%	39	20.7%	18	9.2%	8	13.8%	12	5.7%	5	5.7%	5		
Academic	45.2%	19	23.8%	10	7.1%	3	19.0%	8	4.8%	2	0.0%	0	0.32 (0.751)	(0.056)
Community	44.4%	20	17.8%	8	11.1%	5	8.9%	4	6.7%	3	11.1%	5		
I, or the researchers I work with, have limited access to or knowledge of funding for CEnR	28.7%	25	14.9%	13	9.2%	8	24.1%	21	12.6%	11	10.3%	9		
Academic	28.6%	12	14.3%	6	7.1%	3	33.3%	14	16.7%	7	0.0%	0	1.34 (0.184)	(0.003)
Community	28.9%	13	15.6%	7	11.1%	5	15.6%	7	8.9%	4	20.0%	9		

### Mission and values

The majority of participants across both groups of partners (academic and community) agreed that their institutional mission and value statements demonstrated a commitment to health equity, community/patient-engaged research, and anti-racism. Both academic and community partners always or almost always believed that the institution worked to achieve its mission, vision, and values statement. However, a larger proportion of academic partners (66.7%) than community partners (32.7%) knew if their institution had developed an official institutional public statement recognizing the original, indigenous people as stewards of the physical land where the institution is located (*p* = 0.001). Significant differences were also observed in awareness around institutions’ actions in the service of anti-racism. Among community partners, 38.8% did not know if the AHC with which they were affiliated had mission, vision, or values statements supporting anti-racism, while only 18% of academic partners did not know (*p* = 0.043). Among those who did know, 47.8% of community partners thought the institution always acted to achieve these statements on anti-racism while only 25% of academic partners thought the same (*p* = 0.026). Finally, we saw significant differences between community and academic partners in the degree to which they thought their institutions were working to achieve their health equity goals. Of community partners, 45.2% thought that their institution was always taking action to achieve health equity, while only 23.1% of academic partners thought so (*p* = 0.013).

### Institutional policies and practices

Participants were asked to consider 16 different organizational policies and practices that could facilitate or hinder CEnR/CBPR for AHC research incorporated from previous *E2* survey measures and other administrative and fiscal areas [13], such as IRB practices that were supportive of CEnR/CBPR, involvement of community or patients in stages of research, sharing financial information and providing timely payments to community subcontractors. For many items, large proportions of academic and community participants responded that they *did not know* if their institution had implemented most of the policies and practices (ranging between 18%–50% responded “don’t know” on a given item). However, patterns of academic and community responses differed for all 16 policies/practices when identifying how often policies were applied. Some of the largest differences were observed in the following items: supporting involvement of patient and community partners in all stages of research (community 51% “often,” academic 26.7% “all the time” or “often”; *t* = −3.25, *p* = 0.002); having IRB policies and practices that support CEnR (community reported 47.9% “all the time” or “often,” academic 17.8 %, “all the time” or “often”; *t* = −4.26, *p* < 0.000); identifying and adapting to changes within communities (community reported 56.2% “all the time” or “often,” academic 20.4% “all the time” or “often”; *t* = −3.8, *p* < 0.001), and having community input in strategic planning at the leadership level (community reported 43.7% “all the time” or “often,” academic 8.8%, “all the time” or “often”; *t* = −4.38, *p* < 0.001) (Table 1).

### Community processes and structures

Many academic and community partners did not know to what extent community members and patients are involved in strategic planning for the organization at the top institutional leadership level. Among community partners, 57.4% reported not knowing whether community members or patients were engaged in strategic planning, compared to 35.6% of academic partners (*p* = 0.040).

Community and academic responses differed significantly when asked about the reputation of the institution. Sixty-six percent of community partners and only 40.9% of academic partners agreed (somewhat or strongly) that the institution was recognized for CEnR (*p* = 0.007). In terms of the institutions’ reputation for equity-based research, 59.5% of community partners and 36.4% of academic partners believed their institution had this reputation (*p* = 0.007). Community and academic partners also significantly differed in their belief that community interests were represented at top institutional levels: 60.9% of community partners agreed that their interests were represented and only 38.7% of academic partners felt that community residents’ interests were represented (*p* = 0.002) (Table 2).

### Community Advisory Board (CAB)

Over two-thirds (67%) of study participants reported that the office of community engagement with which they are involved has a CAB. On average, CABs had approximately 20 members, most of whom are community members. Both community and academic participants reported that their CAB engaged to a “great” or “complete extent” in identifying research needs/priorities (46.7% of participants), consulting on cultural issues (56.5%), strengthening collaborations (68.2%), plans for how research findings benefit the community (57.2%), sustainability planning (38.6%), and strategic planning (55.8%). Academic participants identified CAB academic representatives as mostly involved in decision-making related to hiring research personnel, sharing of financial resources, and sharing in-kind resources, where community participants did not know who was mostly involved in these key structural decisions (Table 3).

### Climate and culture for supporting CEnR/CBPR

Overall, the climate and culture of the different institutional sites was assessed to be supportive of CEnR/CBPR. For example, both agreed or strongly agreed that researchers were responsive to patient/community feedback (77.8%), mobilized partnerships to address SDOH factors impacting health outcomes (84.2%), regularly reflected on working towards equity (66.2%), and worked with community partners to support programs and interventions to address health inequities (83.8%). However, researchers and community partners seemed to be faced with having to do this work in a context of scarce resources to support CEnR/CBPR: more than a third of academic partners somewhat or strongly disagreed that sufficient financial resources were present (38.7%) and somewhat or strongly disagreed that training (38.7%) or staffing (54.9%) to support CEnR/CBPR was sufficient. Community participants were significantly more likely than academic partners to agree that training resources (54.6%, *p* = 0.032) and staffing resources (51.5%, *p* = 0.007) were available to support CEnR/CBPR (Table 4).

### Structural racism

These items had a lower response rate than other sections. Of those responded, both academic and community partners reported observing some conduct at the institution that created exclusionary/hostile learning (41.5% academic, 10.5% community, *p* = 0.002).However, 90.3% of community partners and 74.3% of academic partners did not observe unjust promotion, tenure practices (*p* = 0.117); nor did they, on the whole, consider the institutional environment to be exclusionary/hostile to community members/advocates (40% academic, 14.3% community, *p* = 0.025). For the questions presented to only the academic participants, two had a majority of responses: race/ethnicity are important in determining success at their institution (44%), and white scholars have a greater likelihood of success (59%).

### Perceptions of leadership engagement

Institutional leadership can influence climate and culture to support CEnR/CBPR. Community participants (33%) were significantly less likely than academic partners (4.7%) to know if the institutional leadership encouraged or supported investigators to engage in health equity research. However, while academic participants somewhat or strongly agreed that leadership *encouraged* them to engage in health equity research (67.5%), they somewhat or strongly disagreed that leadership *ensured* they had training and development (44.2%) or resources to support it (48.9%). Academic participants were also split about whether leadership supported them to *learn* from their community partners, 33.3% strongly or somewhat disagree and 35.7% somewhat or strongly agree (Table 5).

### Advocacy for CEnR/CBPR

Organizational barriers to implementing CEnR/CBPR are well-known. Since the opportunities to advocate for change within organizations on behalf of community are not always clear, the efforts made by those internal to the organization may not be obvious. Participant responses demonstrated that they collectively agreed (community participants consistently *strongly agreed* and academic participants consistently *somewhat agreed)* that they or the researchers they work with were able to 1) reflect and identify systems of power to influence how community was treated (41% community, 44% academic), 2) negotiate for equitable services on behalf of community (34% community, 38% academic), 3) evaluate efforts to address racial equity issues impacting community (37% community, 51% academic), and 4) develop an action plan to confront barriers to health equity (41% community, 38% academic) (Table 6).

### Perceptions of CEnR/CBPR

Of the many factors that can impact how researchers engage with community partners and organizations, knowledge of who to work with, experience working with partners, and funding to support CEnR are key. The relevance of research to partners was important: academic and community responses *strongly disagreed* (72%) that their research was not relevant to partners. They both *strongly disagreed* that they or the researchers they work with had limited access to knowledge of community partners (community 47% academic 46%,) and limited knowledge and experience engaging partners in research (community 44%, academic 45%). However, responses from participants about having access to or knowledge of funding for CEnR/CBPR were different: academic *somewhat or strongly agreed* (50%) that they did, whereas community participants somewhat or *strongly disagreed* (44.5%) (Table 6).

## Discussion

The IMSS survey results presented represent a convenience sample of academic and community partners from three AHCs engaged in CEnR/CBPR and summarize the perspectives of facilitators and barriers to CEnR/CBPR in their respective locations. To the extent that these results describe similar perspectives of those engaged in CEnR/CBPR in academic environments, the IMSS tool can support identifying specific challenges and priorities for improving CEnR/CBPR capacity. The IMSS tool developed for this study was built on the foundation of evidence from the *E2* body of literature, literature identifying many institutional barriers to CEnR/CBPR [13], and previous survey tools [4,18,19,21]. Based on considerable input and feedback from the champion teams at each participating site and the members of the larger *E2* Think Tank, the research team added additional items to address more complex issues in their respective sites.

These exploratory survey data reinforced several well-known structural supports for CEnR/CBPR by both academic and community respondents. For example, the critical advising role that CABs play in key decision-making related to research was supported by both sample groups [32]. CAB members and the researchers they worked with used their knowledge and experience to advocate for CEnR/CBPR improvements to create a supportive climate for CEnR/CBPR with respect to the research with which they were engaged. Given the convenience sampling of both participant groups with institutional champion teams involved in CEnR/CBPR work, it is possible that the participant responses represented a history of successful collaboration. The type of experience participants have had with CABs (as a researcher, as a CAB member, at the institutional level versus research project level) was not clear and the extent to which CABs are involved in research practices is not clear. While CABs at the institutional level may operate with different functions compared with CABs involved in guiding a specific research project, there is a universal importance to both academic and community partners to prioritize and center voices and perspectives within research that impacts the lives of community members [33].

Understanding the need to advocate for CEnR/CBPR within academic institutions and having the capacity to do so was acknowledged by both academic and community participants. While the presence of CEnR/CBPR continues to expand throughout AHCs, expanded knowledge and skills regarding the nuances of successfully supporting community partners are vital (reflection of systems of power present between partners, negotiating for equitable services and resources, confronting health equity barriers) [34]. As demonstrated in the responses of both group of participants, CEnR/CBPR is critically important to community and patient partners and the communities they represent.

Where academic and community participants’ responses differed was in regard to knowing about and understanding the different institutional structures that might impact their work together. For example, both community and academic participants overwhelmingly responded they *did not know* about the CEnR/CBPR policies/practices applied in their institution (e.g., IRB practices, faculty tenure/promotion, financial practice sharing, review of grant proposals, hiring/training of community, timely payment to partners, written standards of expectations for CEnR/CBPR). This is an important finding for academic participants who might be expected to demonstrate familiarity with their institutions’ research policies and practices.

Academic and community participant responses differed significantly in several critical areas. When asked about whether education was offered to community partners about research, community participants responded it occurred all the time, academic participants responded it was infrequent. Also, community participants responded that community partners are often involved in all stages of research, whereas academic participants stated it was uncommon. Academic participants also did not agree that their institution was recognized for its CEnR/CBPR reputation, whereas community participants were in strong agreement that their institution’s reputation for CEnR/CBPR was well-known. It is understandable that all partners may not be equally aware of the institutional policies and practices impacting CEnR/CBPR. However, the convenience sample of community participants was likely individuals with solid, positive relationships and experiences with individual researchers representing the institution, potentially skewing their favorable perception of the institution. The disparate responses can reflect the differing perspectives of those academic participants internal to the academic institution who are intimately aware of the difficulties stewarding CEnR/CBPR through organizational bureaucracy. This is in contrast to the perspective of community partners who, although experience a positive relationship with an individual faculty member or with the community engagement center, might not be as deeply involved in the project administration internal to the organization [13]. Even more, there are potential differences in how academic and community partners view and experience institutional structures of support for engaging in research. These critical considerations of who is offering their evaluation of the institution and their proximity to the processes involved in CEnR/CBPR would be important to explore in future research with intentional recruitment of community voices who represent a broader mix of experiences and perspectives.

The *I don’t know* (DK) aggregate responses from participants were noticeably higher related to institutional policies and practices (Tables 2 and 4) and staffing and resources (Table 6 and 7), with community responses having higher DK responses. Often DK survey responses are excluded from data analysis or treated as missing data to reduce the threat of validity of study findings [35]. However, when we recognized that the high rates were focused on key structures supportive of CEnR/CBPR in institutions, in consultation with our *E2* partners, we elected to include them to help identify potential targets for institutional change.

The *E2* team shared back the data collected from the IMSS with each champion team in their first-year report. This provided an opportunity for each champion team to review the responses from their academic and community participants and, with *E2* research team members facilitating the conversation, reflect collectively on the meaning of this assessment data and identify the initial targets of change for their institution. For some champion teams, this included prioritizing a greater dissemination of information to community partners and investigators regarding CEnR/CBPR policies and practices.

### Additional data analysis through community reflection

There was an additional opportunity for reflection and analysis of these survey findings when the *E2* Community of Practice met at the end of the project in the spring of 2023. All project site champion teams met in-person along with members of the larger *E2* Think Tank, and UNM *E2* research team members. The aggregate survey findings were presented and discussed in the larger group, and then champion team members were randomly assigned to smaller breakout groups to further discuss how the survey data represented their AHC site, for both the community and academic samples. With this added opportunity to reflect on what we learned from the study findings with the champion teams, we learned that the IMSS tool was helpful to identify the institutional barriers to CBPR/CEnR/PEnR and places to leverage internal (academic participants) and external (community participants) power to create equitable research practices, improve research relationships, and effect institutional research change.

### Limitations

Reflections from both the large and small groups of the champion teams recognized the limitations of this study. One limitation was that the sample of survey participants was recognized as individuals already involved in patient/CEnR and future survey samples needed to include individuals not working with existing research projects. This might explain the findings that community partners believed the institution had better policies and practices because of their own experiences and relationships with individual investigators, whereas academics were more knowledgeable or concerned about what the whole institution was lacking. When addressing the high DK response rates to certain items, champion team members agreed that respondents would not necessarily be aware of institutional policies/practices, staffing, and funding hierarchies [13] and it is difficult to collaborate with research partners to leverage institutional infrastructure.

On the whole, these survey results do not represent all perspectives of CEnR/CBPR at these three institutional sites, nor do they summarize the myriad of challenges of implementing a CEnR/CBPR approach to research in AHC environments. While validated measures from the *E2* Community Engagement Survey were included in the IMSS [4,18], the IMSS was pilot tested for this study with additional measures added to address the aims of the study. The survey results are also characteristic of the sample of academic and community partners from each institutional site who all have different understandings of their respective institutions. We included the DK responses in the analysis results instead of excluding them, as we wanted to increase the analysis power and provide evidence of how CEnR/CBPR institutional context is often unknown by both academic and community participants.

Recruitment for survey participation was challenging for each champion team reflected by the low response rate to the survey and to specific survey questions. Feedback from champion team members indicated that the length of the survey itself was a burden to participants which may have contributed to the missing responses. The differences in institutional contexts also need to be considered when comparing results between institutional sites.

These reflections facilitated discussion of suggested changes for the IMSS, including broadening the sample and directly addressing exclusionary and inclusionary language. Recommendations for the IMSS included adding new questions about: what does partnership mean to community partners; is partnership always beneficial; questions specific for populations (Native American, Latino/x, Black, LGBTQ+, etc.); ideas for practices or work-arounds for institutional barriers (e.g., paying community partners); and identifying the data needs and research capacities of community and patient organizations. Survey items for existing assessment questionnaires administered to community members (e.g., how community partners view the institution, its reputation, and responsiveness to community concerns) were suggested to increase community responses and decrease survey burden.

## Conclusion

The PCORI engagement award enabled the first pilot testing of our IMMS tool. We found that the IMSS tool can be used to prompt the necessary reflection of CEnR/CBPR/PEnR partners within and external to academic institutions to address barriers to authentic engagement, and prompt partners’ inclusion of leadership within AHCs and communities to increase institutional support for CEnR/CBPR. Using the *E2* tools of data-focused collective reflection, the data collected with the IMSS tool can be a mechanism to spur a movement toward identifying and addressing the institutional barriers and facilitators of sustained CEnR/CBPR experienced by individuals who are both external and internal to the organization. With our new PCORI Science of Engagement award, the E2 team will revise the IMSS survey tool for testing and validation with a different and larger sample of eight AHCs. This will allow for psychometric testing of measurement scales, potential analyses of data from academic versus community participants, or in response to questions generated with our partner institutions. These crucial steps will be used to meet the goals for community engagement at AHCs and provide the needed bridge between the expressed desire by researchers and leaders of academic and research institutions to authentically engage the community with whom they serve and to seek to strengthen equitable and supportive practices for engaged research [36].
